# Fas-associated protein with death domain (FADD) regulates autophagy through promoting the expression of Ras homolog enriched in brain (Rheb) in human breast adenocarcinoma cells

**DOI:** 10.18632/oncotarget.8249

**Published:** 2016-03-22

**Authors:** Liangqiang He, Yongzhe Ren, Qianqian Zheng, Lu Wang, Yueyang Lai, Shengwen Guan, Xiaoxin Zhang, Rong Zhang, Jie Wang, Dianhua Chen, Yunwen Yang, Hongqin Zhuang, Wei Cheng, Jing Zhang, Zi-chun Hua

**Affiliations:** ^1^ The State Key Laboratory of Pharmaceutical Biotechnology, School of Life Sciences, Nanjing University, Nanjing, 210023, Jiangsu, China; ^2^ Changzhou High-Tech Research Institute of Nanjing University and Jiangsu Target Pharma Laboratories Inc., Changzhou, 213164, Jiangsu, China

**Keywords:** FADD, Rheb, autophagy, tumorigenesis, breast cancer

## Abstract

FADD (Fas-associated protein with death domain) is a classical adaptor protein in apoptosis. Increasing evidences have shown that FADD is also implicated in cell cycle progression, proliferation and tumorigenesis. The role of FADD in cancer remains largely unexplored. In this study, *In Silico* Analysis using Oncomine and Kaplan Meier plotter revealed that FADD is significantly up-regulated in breast cancer tissues and closely associated with a poor prognosis in patients with breast cancer. To better understanding the FADD functions in breast cancer, we performed proteomics analysis by LC-MS/MS detection and found that Rheb–mTORC1 pathway was dysregulated in MCF-7 cells when FADD knockdown. The mTORC1 pathway is a key regulator in many processes, including cell growth, metabolism and autophagy. Here, FADD interference down-regulated Rheb expression and repressed mTORC1 activity in breast cancer cell lines. The autophagy was induced by FADD deficiency in MCF7 or MDA-231 cells but rescued by recovering Rheb expression. Similarly, growth defect in FADD-knockdown cells was also restored by Rheb overexpression. These findings implied a novel role of FADD in tumor progression via Rheb–mTORC1 pathway in breast cancer.

## INTRODUCTION

Fas-associated protein with death domain (FADD) is the key adaptor protein transmitting apoptotic signals mediated by death receptors (DRs). It was originally identified in FAS-induced apoptosis [1–3]. Following DD interaction between FADD and FAS, the cytoplasmic procapase-8 binds to FADD through DED-DED interactions, and forms the death-inducing signaling complex (DISC). Besides being a main death adaptor molecule, FADD is also required for T cell proliferation. Several groups have demonstrated that FADD deficiency in peripheral T lymphocytes resulted in an inhibition of mitogen-induced T cell proliferation [4, 5]. FADD deficiency also leads to a dysregulation of the cell cycle machinery. Recently, emerging evidences have shown that FADD expression was associated with tumor development [6]. Amplification of the 11q13, a chromosomal region containing the gene encoding FADD, is frequently observed in many cancer cells. Overexpression of FADD might be as a biomarker in head and neck squamous cell carcinoma [7, 8]. FADD protein expression could contribute to disease progression in several malignancies, so the mechanism of FADD in tumorigenesis needs further investigated.

At present, *In Silico* Analysis using Oncomine Database is a useful platform to gain the disease summary for FADD, and proteomics coupled with bioinformatics analysis provides a powerful tool for us to find the potential targets of FADD and its signaling pathway networks. In this study, we first reported that FADD expression was remarkably higher in breast cancer and applied LC-MS/MS detection plus bioinformatics analysis to reveal that Rheb-mTORC1 pathway was dysregulated in breast cancer cells because of FADD knockdown. mTOR is a serine/ threonine kinase and functions as a key modulator in cell proliferation, protein synthesis, aging and autophagy [9, 10]. The best-described target of mTORC1 is its downstream marker ribosomal S6 protein kinase 1 (p70s6k). p70s6k activation requires mTORC1-mediated phosphorylation [11]. The mTORC1 activity is tightly regulated by a wide range of environmental signals. One key upstream activator of mTORC1 is the small GTP-binding protein Rheb (Ras homolog enriched in brain), which is the most well-known regulator of mTORC1 to date. Rheb promotes mTORC1 activity and enhances p70s6k phosphorylation in a rapamycin-dependent manner [12–15]. Latest studies show that Rheb-mTORC1 signaling axis is hyper-activated in a variety of human cancers and closely related to tumorigenesis [16, 17].

Therefore, we performed further cell biological examinations on FADD knockdown to address the Rheb-mTORC1 pathway. Our data showed that FADD interference decreased Rheb expression on the transcriptional level. To explore the effect of FADD on Rheb-mTORC1 signaling axis, we detected the p70s6k phosphorylation for mTOR activity. The decrease of p70s6k phosphorylation was observed in FADD knockdown cells, which was rescued by recovered Rheb expression. Inhibition of autophagy is one important function of mTORC1. Similarly, the induction of autophagy by FADD deficiency was also rescued by recovering Rheb expression. Moreover, Rheb overexpression could improve cell growth which was retarded for FADD knockdown. Collectively, these data suggest a novel role of FADD in breast tumorigenesis through promoting Rheb expression.

## RESULTS

### High expression of FADD in human breast cancer correlated with poor prognosis

**Figure 1:**
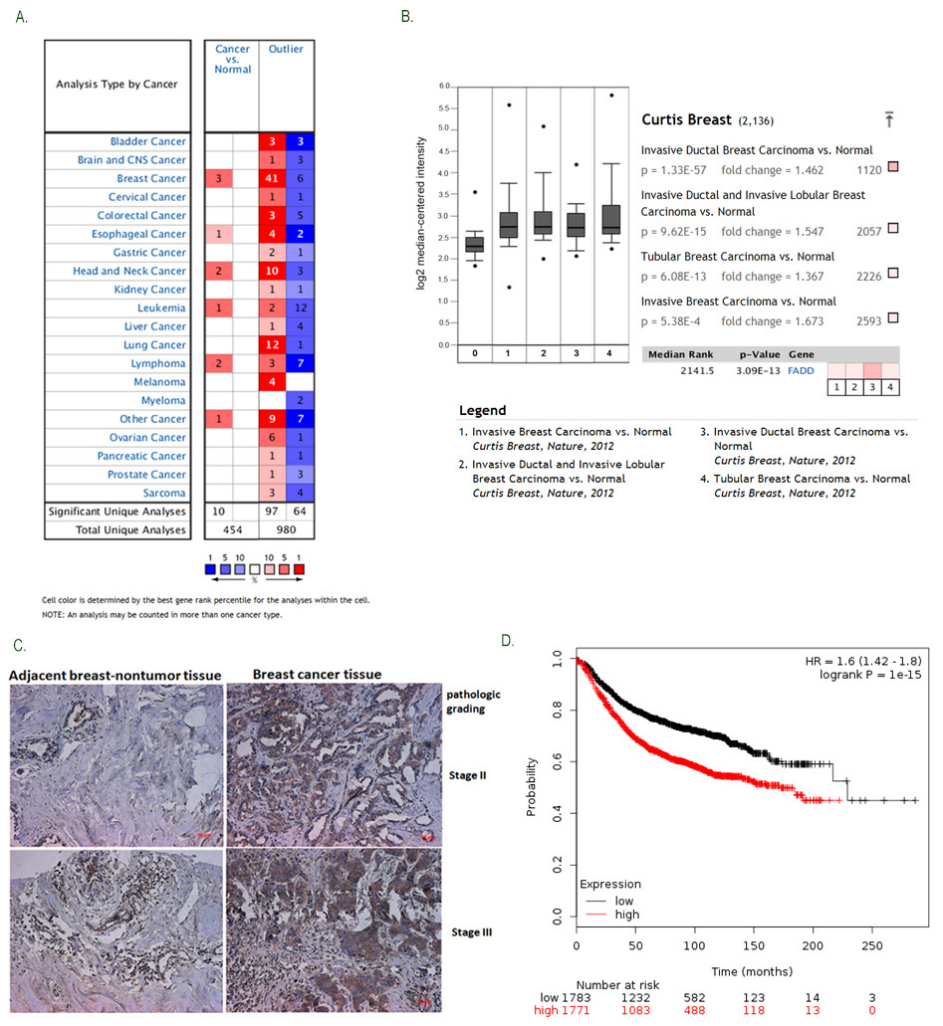
Elevated FADD expression was correlated with human breast cancer progression **A.** The gene summary for FADD in cancers was gained from OncomineTM Research Edition. **B.** Increased FADD mRNA expression was analyzed in Curtis Breast dataset with 2,136 samples. **C.** FADD expression was determined by immunohistochemical analysis on tissue microarray. Representative images (pathological grades: II and III) were shown: (a) adjacent histologically nontumor tissue, (b) breast tumor tissue. **D.** Cancer survival analysis of FADD expression was assessed on Kaplan-Meier plotter. Meta-analysis based on biomarker assessment shows that High FADD expression versus low expression has a poor survival in human breast cancer. P-value is calculated using log-rank test.

Oncomine platform (http://www.oncomine.org) is a free online bioinformatic resource of cancer transcriptome data. To gain an overview of FADD expression in human cancers, we performed analysis of published patient data using Oncomine and found that FADD mRNA level is significantly up-regulated in human breast cancer (Figure 1A). In Curtis breast dataset with 2136 samples [18], FADD expression levels were upregulated in most of breast cancer tissues (n>1556, p=3.09E-13), compared with normal tissues (n=144) (Figure 1B). To confirm the oncomine data, we analyzed FADD expression in a breast tissue microarray (TMA) containing 30 cases of breast specimens by Immunohistochemical (IHC) staining (Figure 1C). High FADD expression was observed in 21 of 30 (70%) of tumor tissues compared with adjacent histologically normal tissues, suggesting elevated FADD expression might contribute to tumor development. Using Kaplan Meier plotter, another free online tool for meta-analysis based biomarker assessment [19], the result revealed that FADD-High expression in patients was correlated with a worse survival ratio compared with FADD-low counterparts (HR=1.6, logrank P=1e-15) (Figure 1D). Collectively, these findings indicate that up-regulated FADD predicts a poor prognosis in breast patients and is closely correlated with tumor progression in breast cancer.

### LC-MS/MS based proteomics analysis in breast cancer cell

**Figure 2:**
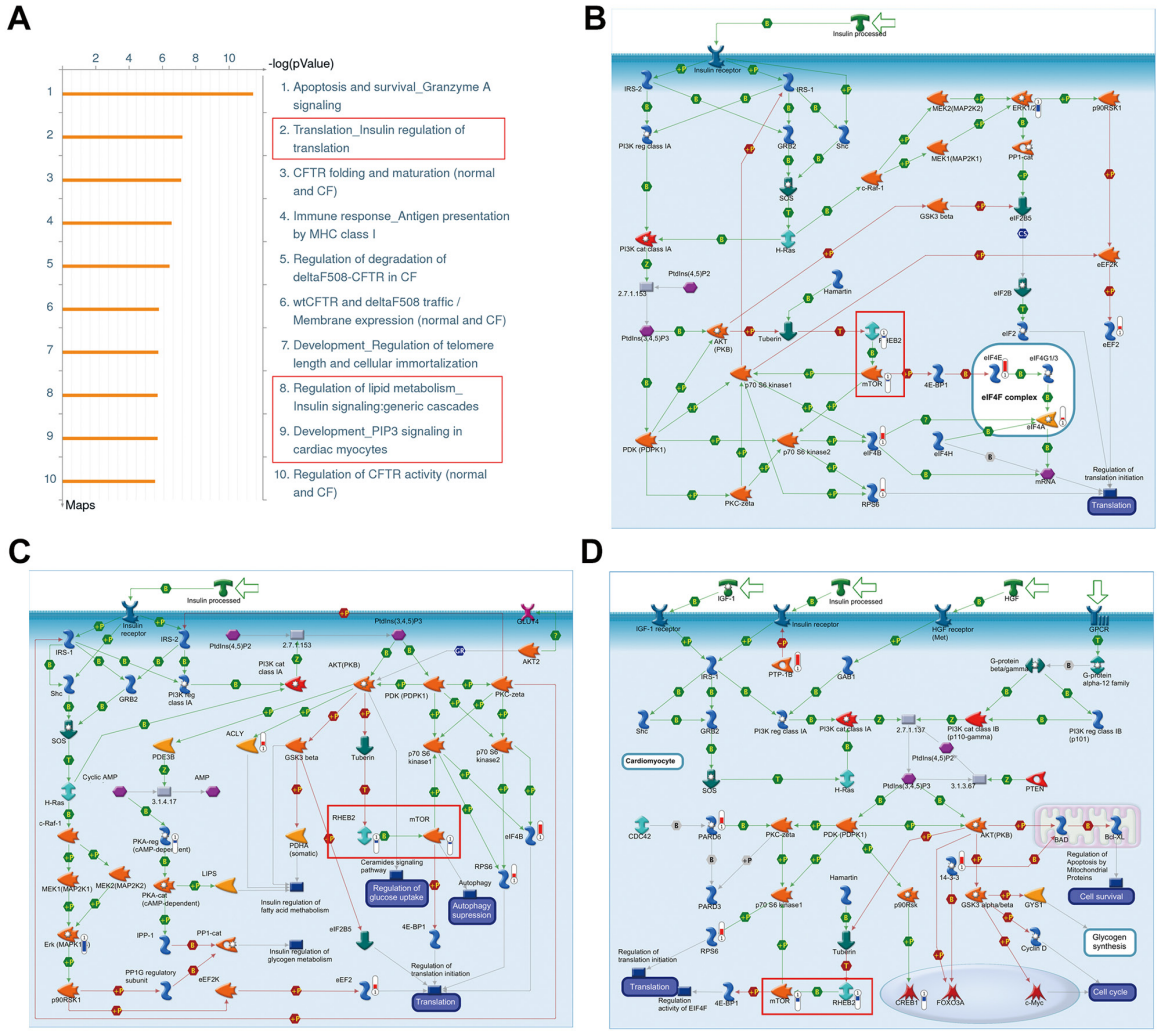
Enrichment analysis of differentially expressed proteins in MCF-7 cells with FADD knockdown **A.** Top ten most significant pathways predicted by GeneGo pathway analysis. The results were ordered by -log10 of the p value of the hypergeometric distribution. **B–D.** Pathways related to Rheb-mTORC1 signaling axis within the top ten most significant pathways. Thermometers with blueorred next to symbols show proteins identified in LC-MS/MS detection: red color represents the proteins that increased in FADD knockdown MCF-7 cells compared to control group; blue color represents the decreased proteins.

To find out the molecular pathways directly or indirectly controlled by FADD in tumorigenesis of breast cancer, high throughput proteomic approaches was performed in human breast cell line MCF-7 with FADD knockdown. The expression of FADD was confirmed by western blotting shown in Supplementary Figure S1A. About 500 differentially expressed proteins were identified. We used the GeneGO/MetaCore software to analyze the biological networks related to these proteins. GeneGo Map Folder analysis was applied to predict top ten pathways in the highest significance in Figure 2A. Pathway in apoptosis and survival ranked first, which was consistent with the main function of FADD as an apoptotic protein. Among them, three pathways were linked with Rheb-mTORC1 signaling axis (Figures 2B–2D). Further analysis on GeneGo process also showed that three of the top ten processes were linked to Rheb-mTORC1 signal axis (Supplementary Figure S1B). Since mTOR pathway is a key regulator of cell growth and proliferation, its deregulation might be an important clue for FADD function in breast cancer.

### Rheb expression decreased by FADD knockdown

**Figure 3:**
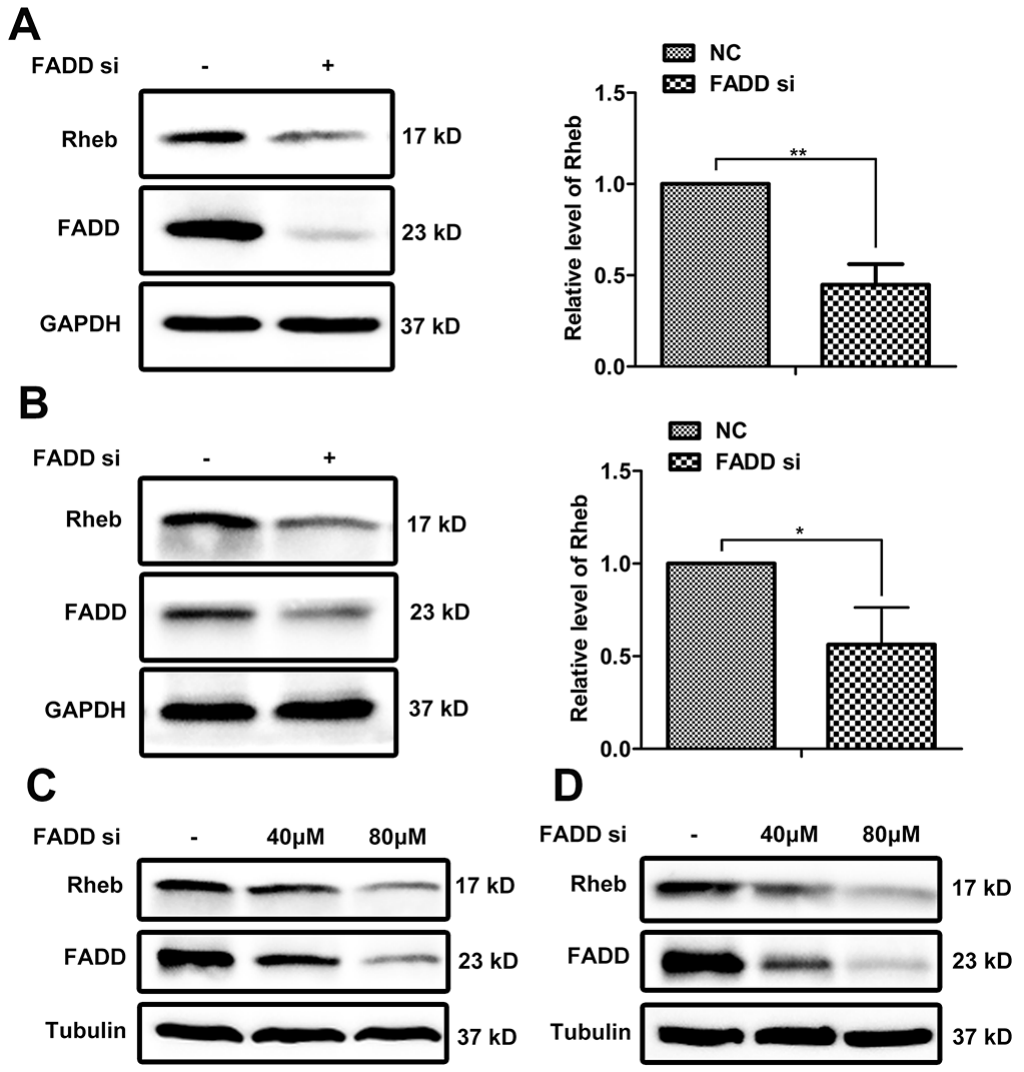
FADD interference downregulated Rheb expression NC or FADD siRNA was transfected in MCF-7 **A.** or MDA-MB-231 **B.** cells for 48 h. Total cell lysates were detected by western blotting with antibodies as indicated. Band intensity was quantified by chemiAnalysi software. Data was represented as mean ± S.D. P values were calculated by Student's t-test (*p < 0.05; **p < 0.01) and the experiment was repeated at least three times. Different dose of FADD siRNA was transfected into MCF-7 cells **C.** and MDA-MB-231 cells **D.** The expression of FADD and Rheb was detected by western blot.

Proteomics analysis showed that the expressions of Rheb and mTOR were down-regulated in MCF-7 cells of FADD knockdown compared with control cells (Supplementary Table S1), which was confirmed by western blotting analysis (Figure 3A). There was about 60% reduction of Rheb protein in MCF-7 cells treated with FADD siRNAs. No significant difference on mTOR expression was observed (Supplementary Figure S2). Similar result was reconfirmed in another breast cancer cell line MDA-MB-231 (Figure 3B). With an increasing transfection of FADD siRNAs, the protein level of Rheb was decreased in a dose-dependent manner in both MCF-7 and MDA-MB-231 cells (Figure 3C and 3D). Notably, Rheb expression was also elevated in breast TMA as well as FADD (Supplementary Figure S3), and reported to be correlated with poor prognosis in patients with breast cancer [20]. The protein level of Rheb had a good consistency with FADD expression in breast cancers.

### The effect of FADD on Rheb transcription

**Figure 4:**
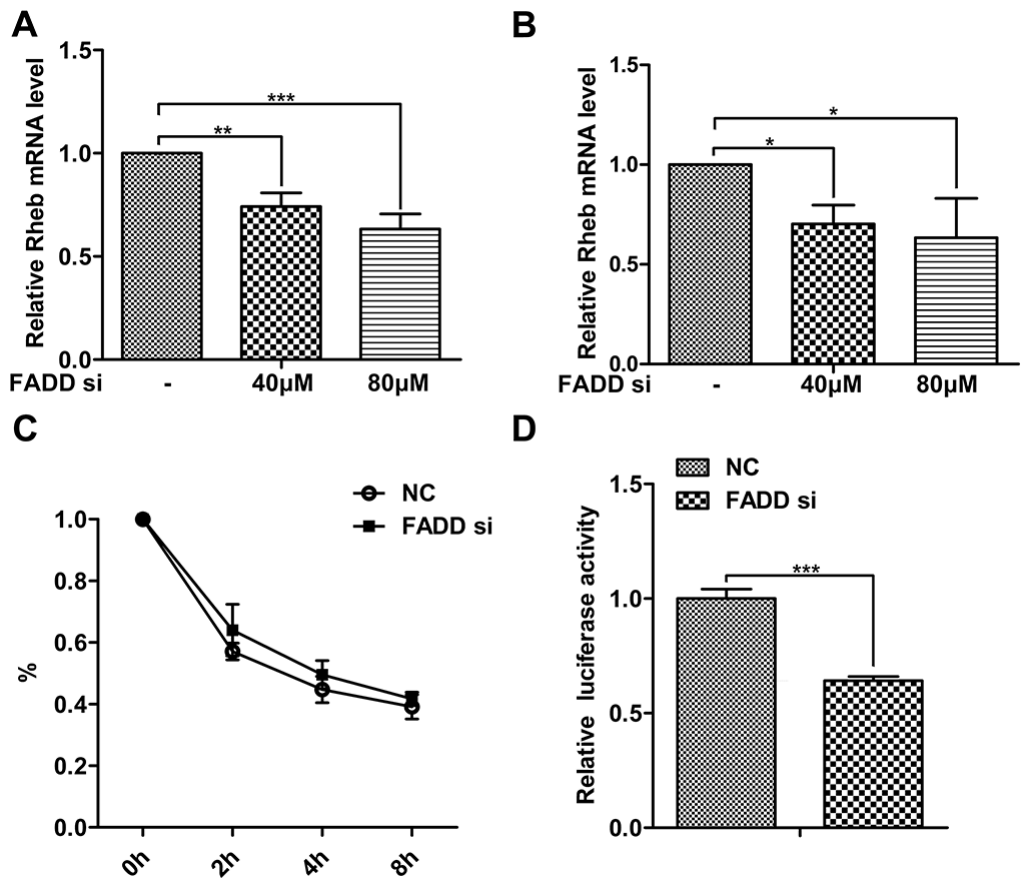
FADD interference decreased Rheb expression on the transcriptional level FADD siRNA was transfected into MCF-7 **A.** and MDA-MB-231 **B.** cells for 48 h. FADD mRNA was measured by qRT-PCR normalized to β-actin. **C.** MCF-7 cells were transfected with 80 μM FADD siRNA/NC for 48 h and then treated with 5 μg/ml Act D for indicated time. Rheb mRNA were quantified by qRT-PCR. **D.** Luciferase activity of Rheb-promoter reporter was detected in MCF-7 cells transfected with FADD siRNA/NC. Each bar is the mean of at least 3 independent experiments. Data are representedas mean ± S.D. *p < 0.05; **p < 0.01;***p < 0.001.

We next tested the effect of FADD on Rheb gene expression at the transcription level. After RNAi for FADD, Rheb mRNA was examined by qPCR assays in MCF-7 cells (Figure 4A) and MDA-MB-231 (Figure 4B). Consistent with the above data, Rheb mRNA was also decreased in a dose dependent manner when FADD became gradually reduced. The decrease of mRNA level is generally considered for two factors, mRNA stability and transcriptional activity. To examine the effect of FADD on the stability of Rheb mRNA, MCF-7 cells were transfected with FADD siRNA or control siRNA for 48 h and treated with actinomycin D (ActD) for indicated times, then harvested to quantify the Rheb mRNA by qPCR. As shown in Figure 4C, the degradation speed of Rheb mRNA showed no obvious differences in two groups. Then we constructed the promoter-luciferase reporter vector to analyze the transcriptional activity of Rheb. FADD interference inhibited the luciferase acitivity of Rheb promoter (Figure 4D), suggesting the effect of FADD on Rheb expression at its transcriptional level.

### mTORC1 activity regulated by FADD through Rheb

**Figure 5:**
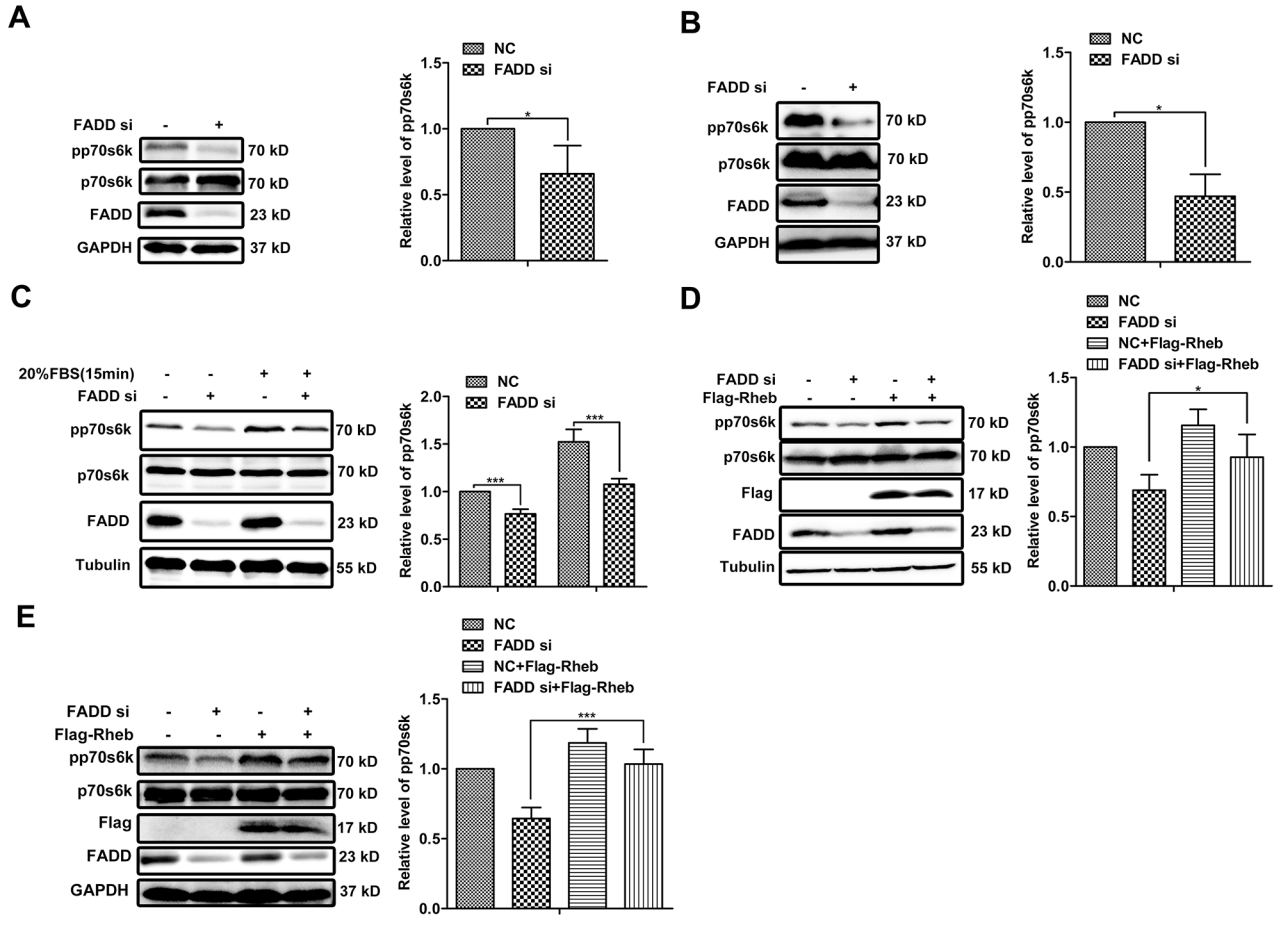
FADD regulated mTORC1 activity through Rheb MCF-7 **A.** or MDA-MB-231 **B.** cells were transfected with FADD siRNA/NC for 48 h. The cell lysates was performed by western blot with indicated antibodies (n=4). **C.** MCF-7 cells were transfected with FADD siRNA/NC for 24 h and then starved for 24 h. Then cells were stimulated by 20% serum for 15 minutes (n=3). Cells were cotransfected with FADD siRNA/NC with pRK5-Flag-Rheb or control vector for 48 h. Western blot analysis in MCF-7 **D.** or MDA-MB-231 **E.** Band intensity was quantified by chemiAnalysi software. Data are represented as mean ± S.D. *p < 0.05; ***p < 0.001. Each bar is the mean of 3 independent experiments.

Based on GeneGo Map analysis, Rheb-mTORC1 signaling axis became unusual because of FADD knockdown. p70s6k is a well-defined downstream of mTORC1 and its phosphorylation is a reliable measurement for mTORC1 activity [11, 15, 21]. Compared with transfection with NC siRNA, the level of p70s6k phosphorylation decreased to 60% in MCF-7 cells transfected with FADD siRNA (Figure 5A) and 40% in MDA-MB-231 cells (Figure 5B), respectively. The mTORC1 activity is tightly regulated by a wide range of environmental signals, including serum. MCF-7 cells were transfected with FADD siRNA for 24 h and then starved with DMEM without serum for 24 h followed by 20% serum stimulation for 15 min [22]. The stimulation of serum effectively enhanced p70s6k phosphorylation in MCF-7 cells with control siRNA, and no much phosphorylation shown in cells with FADD siRNA (Figure 5C), indicating an impairment of mTORC1 activity in FADD deficiency. To examine whether the influence of FADD on the mTORC1 activity is mediated by Rheb, we recovered Rheb expression in FADD-knockdown-MCF-7 cells via transfecting Rheb expression vector. p70s6k phosphorylation was rescued from 68% to 80% by supplement of Rheb expression (Figure 5D), which was also observed in MDA-MB-231 cells (Figure 5E). These findings suggested that Rheb was necessary for FADD modulation on mTORC1 activity.

### Autophagy induced by FADD silencing in human breast cancer cells

**Figure 6:**
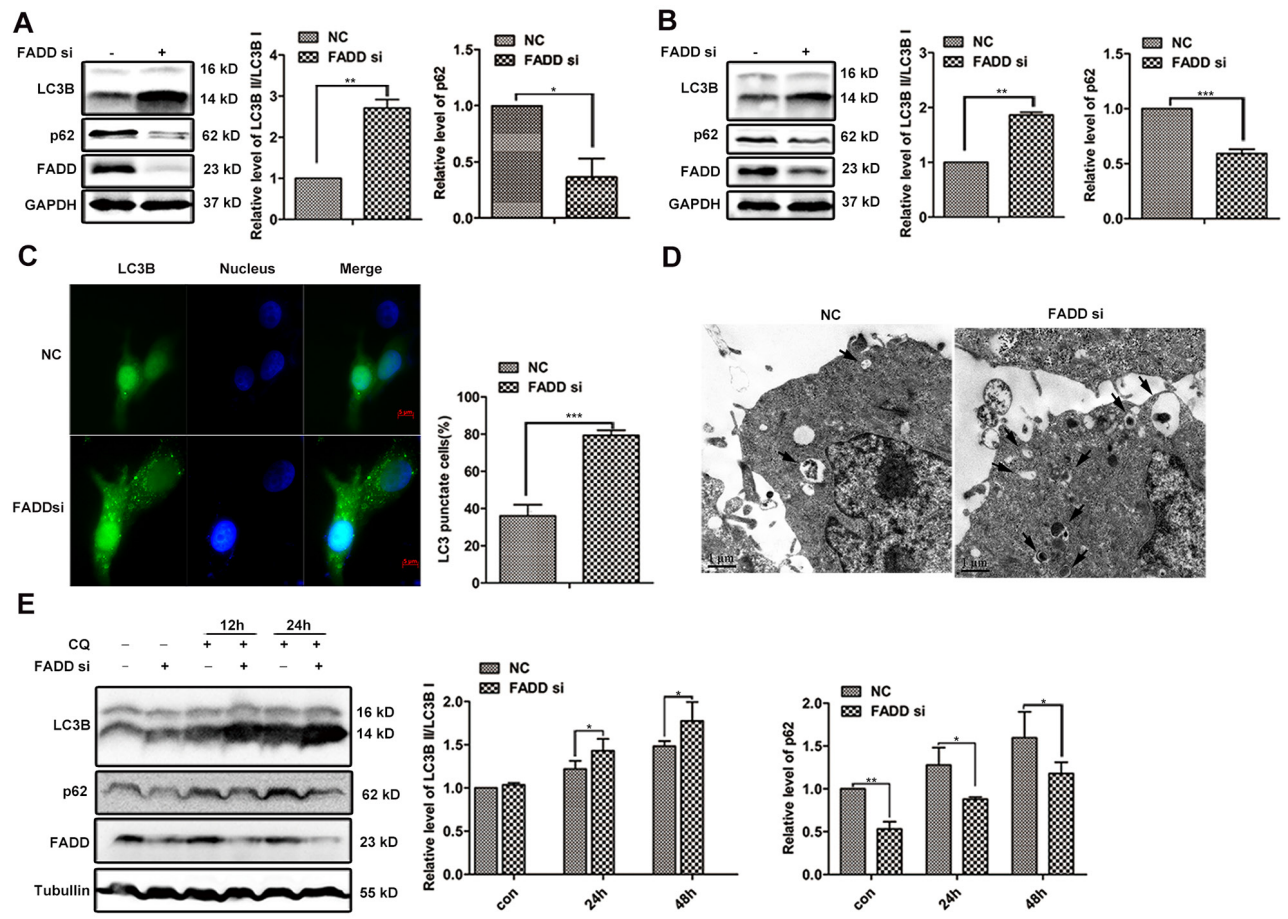
FADD interference induces autophagy MCF-7 **A.** or MDA-MB-231 **B.** cells were transfected with FADD siRNA/NC for 48 h. LC3B and p62 were detected by western blotting. Band intensity was quantified. Each bar is the mean of 4 independent experiments. **C.** MCF-7 cells were transfected with GFP-LC3 plasmid together with FADD siRNA/NC for 48 h. Formation of vacuoles containing GFP-LC3 (dots) was examined by fluorescence microscopy. Scale bar: 5 μm. **D.** MCF-7 cells were transfected with FADD siRNA/NC for 48 h and then conducted to transmission electron microscopy (TEM) analysis. Representative image of different experiment was shown. Scale bar: 1 μm. **E.** MCF-7 cells were transfected with FADD siRNA/NC for 24 h and then treated with 20 μM chloroquine (CQ) at indicated times. Cell lysates were detected by western blotting (n=3). Each bar is the mean of 3 independent experiments. Data are represented as mean ± S.D. *p < 0.05; **p < 0.01; ***p < 0.001.

One important function of mTORC1 is the inhibition of autophagy [23–25]. Considering the impairment of mTORC1 activity in FADD deficiency cells might initiate autophagy, we next detected autophagy using LC3B as a marker. During autophagy, LC3B I will be modified with phosphatidylethanolamine (PE) and converted to LC3B II, and the ratio of LC3B II to LC3BI is widely used to measure cellular autophagic activity [26, 27]. LC3BI to LC3BII conversion was markedly increased in MCF-7 or MDA-MA-231 cells when FADD was knocked down (Figure 6A and 6B). Meanwhile, another autophagosomal marker p62 expression was observed in 40% to 60% reduction accompanied by FADD reduction, which is reported to degrade during autophagy. When autophagy was induced by starvation, stronger autophagy activity was also observed in cells of FADD knockdown (Supplementary Figure S4). For avoiding the artificial effect, we tried another interference technique Crispr/Cas9 to effectively downregulate FADD expression and obtained similar results (Supplementary Figure S5). Furthermore, GFP-LC3 was used to display the images of autophagy. The formation of GFP-LC3 labeled vacuoles increased significantly in MCF-7 cells with deficient FADD, and the quantitation of GFP-LC3-punctate cells were shown in Figure 6C. Furthermore, autophagy induced by FADD silencing was confirmed by the morphological change using transmission electron microscopy (TEM) analysis (Figure 6D). There were more double-membrane cytoplasmic vacuoles (arrowheads) in cells transfected FADD siRNA than control siRNA, indicating the stronger autophagic activity. Chloroquine (CQ) inhibits autophagy as it leads to inhibition of both fusion of autophagosome with lysosome and lysosomal protein degradation. CQ treatment resulted in the accumulation of LC3B and p62, but did not change the appearance of the more conversion of LC3B I to LC3B II and lower expression of p62 in FADD-knockdown cells. These data demonstrated that FADD interference promoted the occurrence of autophagy in the early stage.

### The autophagy mediated by FADD via Rheb-mTOR pathway

**Figure 7:**
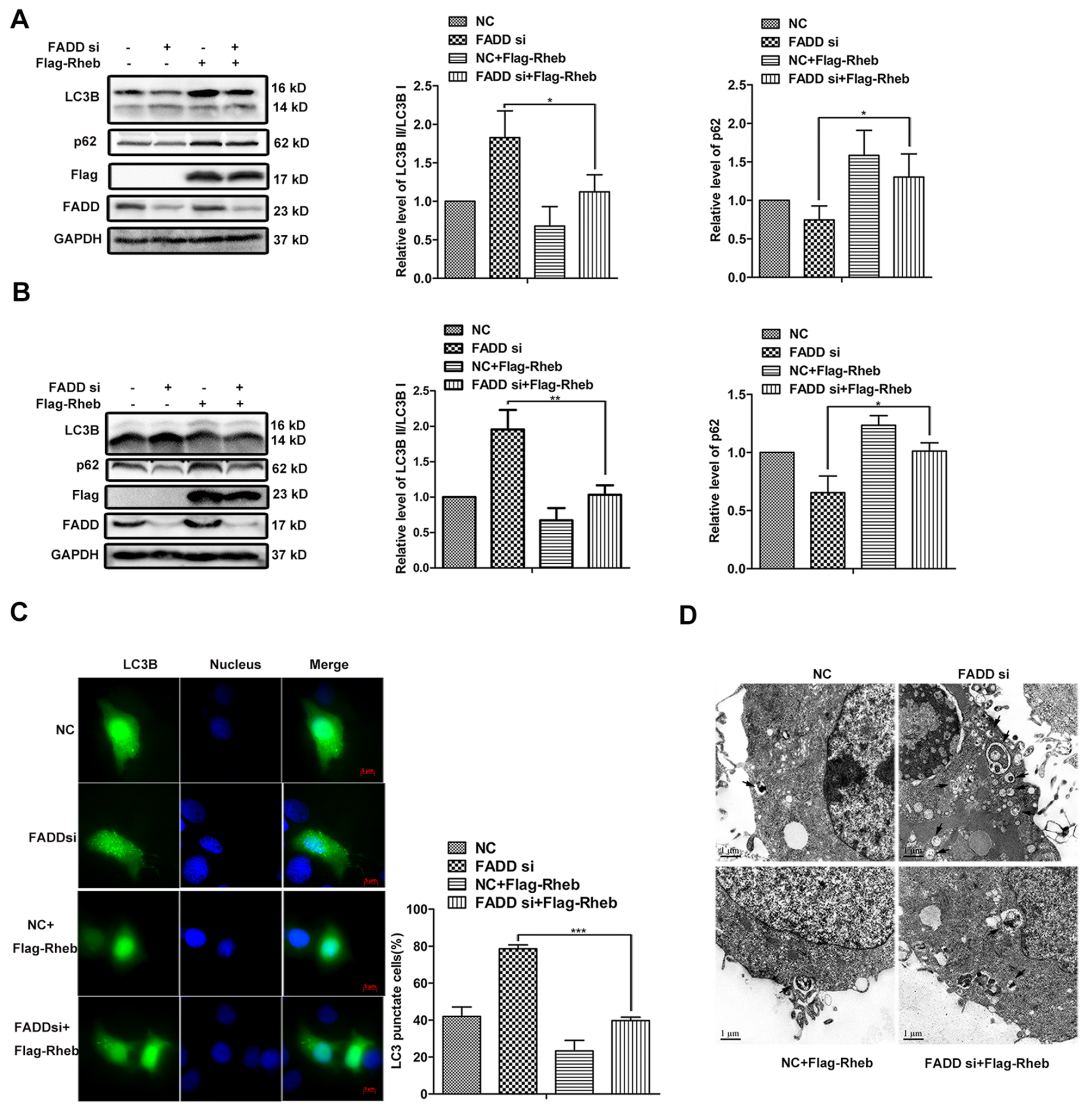
Rheb overexpression rescued autophagy induced by FADD knockdown MCF-7 **A.** or MDA-MB-231 **B.** cells were transfected with FADD siRNA/NC together with pRK5-Flag-Rheb or control plasmid for 48 h and then lysed for western blotting. Band intensity was quantified. Each bar is the mean of 3 independent experiments. **C.** MCF-7 cells were cotransfected GFP-LC3 with FADD siRNA/NC, pRK5-Flag-Rheb, control vector as indicated. Formation of vacuoles containing GFP-LC3 (dots) was examined by fluorescence microscopy. Scale bar: 5 μm. **D.** MCF-7 cells were transfected with FADD siRNA/NC together with pRK5-Flag-Rheb/control plasmid for 48 h and then subjected to transmission electron microscopy (TEM) analysis. The representative of three different experiments was shown. Scale bar: 1μm. Data are represented as mean ± S.D. *p < 0.05; ***p < 0.001.

To examine whether Rheb is necessary for FADD-mediated autophagy, Rheb expression vector was cotransfected with FADD siRNA or control siRNA, respectively. The ratio of LC3B II/LC3B I was declined and p62 expression was increased when Rheb overexpression in both MCF-7 cells (Figure 7A) and MDA-MB-231 cells (Figure 7B). By fluorescence image, we observed that both the number of dots inside cells and the percentage of cell with GFP-LC3 puncta-formation were decreased because of Rheb overexpression, especially in FADD knockdown cells (Figure 7C). Transmission electron microscopy analysis further revealed that the autophagosome formation mediated FADD interference was inhibited by Rheb overexpression (Figure 7D). These data provided evidences for the role of FADD in autophagy via Rheb-mTOR pathway.

### The crosstalk of cell proliferation and autophagy linked by FADD

**Figure 8:**
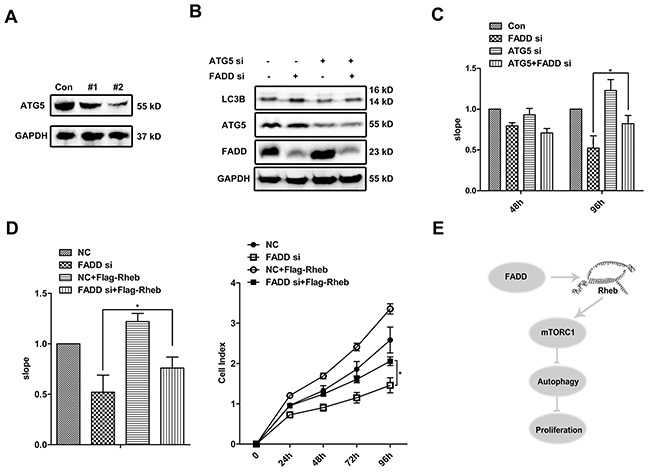
FADD enhances cell proliferation by repressing autophagy **A.** ATG5 siRNAs/NC was transfected in MCF-7 cells for 48 h. ATG5 expression was detected. **B.** MCF-7 cells were transfected with ATG5 siRNA/NC together with FADD siRNA/NC for 48 h and detected by western blotting. **C.** MCF-7 cells were transfected with ATG5 siRNA/NC together with FADD siRNA/NC and then seeded to E-Plate on Real-Time Cell Analysis (RTCA). Each bar is the mean of 4 independent experiments. Data are representedas mean ± S.D.*p < 0.05. **D.** MCF-7 cells were transfected with FADD siRNA/NC together with pRK5-Flag-Rheb and then performed to RTCA analysis. Each bar is the mean of 4 independent experiments. Data are represented as mean ± S.D.*p < 0.05. **E.** Proposed model of FADD in autophagy.

The mTOR pathway integrates signals from nutrients and growth factors to regulate many progresses, including autophagy and cell proliferation. It interested us to figure out the influence of FADD on these two progresses. Two siRNAs of ATG5 was designed for blocking autophagy at early stage. #2 ATG5 siRNA seemed a more effective candidate and was thus used in later experiments (Figure 8A). As expected, ATG5 interference reduced the autophagic activity (Figure 8B). Then we monitored cell growth using Real-Time Cell Analysis (RTCA), which is a novel approach to assess cellular proliferation. The slope processed by software represents the growth rate showed in Figure 8C. At 48 h after transfection, there was no significant difference on cell proliferation among four groups. Extended to 96 h post transfection, the growth of cells treated with FADD siRNA was obviously restrained. Meanwhile when cells treated with double siRNAs of FADD and ATG5, it showed partly restored in cell proliferation (Figure 8C). For continuous culture for 96 h without supplement of fresh culture medium, nutrient deficiency would induce autophagy. At this time, the defect on cell proliferation by FADD siRNA was partly rescued by addition of ATG5 siRNA, suggesting that autophagy induced by FADD deficiency might be one important reason for cell growth defect. We further examined whether Rheb overexpression would effectively recover the impairment of cell proliferation mediated by FADD deficiency. Consistent with the previous results, Rheb also improved cell proliferation of FADD-deficient cells (Figure 8D).

## DISCUSSION

Recently, amplification of FADD has been observed in many different types of cancer and links to cancer progression [28–31]. Here, we provided evidence that FADD overexpression correlated with poor outcome in human breast cancer for the first time. With the help of high-throughput proteomics and bioinformatics analysis, the Rheb-mTORC1 pathway was predicted to be dysregulated in human breast adenocarcinoma cell line MCF-7 when FADD was knockdown (Figure 2). Rheb has been regarded as a novel prognostic factor in human cancer for that it activates the key metabolic regulator mTORC1. Elevated Rheb expression has been reported in a wide variety of tumors and coupled with mTORC1 hyper-activation, including human breast cancers [34, 57, 58]. In our study, Rheb downreglation by FADD deficiency was validated in human breast cancer cell lines MCF-7 and MDA-MB-231 (Figure 3), as well as the impairment of mTORC1 activity (Figure 5). Like FADD, high Rheb expression is also correlated to poor prognosis in human breast cancer [20].

FADD is much more than an instrument of death and implicated in embryonic development, cell proliferation, tumor progression, inflammation, necrosis, and autophagy. However, the most important function of FADD is a pro-apoptotic adaptor. We previously reported that FADD protein had the potential to highly oligomerize. FADD self-aggregated in vitro and transfected FADD in mammalian cells effectively induced apoptosis by forming death effector filaments independent of receptor cross-linking at the plasma membrane [40]. The apoptosis induced by FADD overexpression also appeared in breast cancer cells (shown in Supplementary Figure S6), so RNA interference of FADD as a reasonable and practical approach of studying the effect of FADD protein expression level was widely used in our studies. In order to consolidate this conclusion of Rheb expression regulated by FADD, the results were fully verified in FADD-knockout MEF cells shown in Supplementary Figure S7. FADD could up-regulate Rheb expression and activate mTORC1 activity.

The mTORC1 activity is tightly related with cellular processes like autophagy and cell proliferation [32]. FADD interference induced autophagy by down-regulating Rheb-mTORC1 activity, which was restored by recovering Rheb expression. Similarly, the proliferative deficiency caused by FADD silencing was also rescued by Rheb overexpression. Our findings indicated that Rheb might play an important role in the function of FADD on tumorigenesis.

Growing evidence has shed light on the role of autophagy in proliferation and tumorigenesis. ATG5−/−CD4+ and CD8+ T cells failed to undergo efficient proliferation after TCR stimulation [33]. However, unrestricted autophagy impairs cell proliferation [34, 35]. Mice with systemic deletion of ATG5 and liver-specific ATG7−/−mice were reported to develop benign liver adenomas, together with elevated cell proliferation [36]. Similarly, ATG5 interference inhibited autophagy and partly rescued the proliferative deficiency in FADD knockdown cells. This might be a possible way in the proliferative role of FADD via regulating autophagy.

In conclusion, our study strengthened the role of FADD in human breast tumorigenesis. FADD upregulates Rheb expression and promotes mTORC1 activity. Activated mTORC1 augments cell proliferation via autophagy inhibition. This finding helps to enrich the multifunction of FADD, and more importantly, represent a promising target for breast cancer therapy.

## MATERIALS AND METHODS

### Plasmids and reagents

The encoding sequence of human Rheb was amplified from cDNA of MCF-7 cells and cloned into pRK5-Flag vector. The promoter sequence of human Rheb (−1064/+217) was amplified and cloned into pGL3 reporter vector. GFP-LC3 plasmid was purchased from Addgene (#24987). Actinomycin D (Act D, 01815), chloroquine (CQ, C6628) were purchased from Sigma. Hoechst 33342 (H3570) was from Invitrogen.

### Cell culture

Breast adenocarcinoma cell lines MCF-7 and MDA-MB-231 were cultured in Dulbecco's modified Eagle's medium (Wisent, Canada) supplemented with 10% fetal bovine serum (Invitrogen, USA), 50 μg/ml streptomycin and 50 U/ml penicillin. Cells were kept at 37¼C in a humidified incubator with 5% CO2.

### siRNAs and transfection

All synthetic siRNAs and the negative control (NC) were purchased from Shanghai GenePharma Co. Ltd. For transfection, cells were transiently transfected with siRNAs or plasmids using lipofectamine 2000 (Invitrogen, USA) according to the manufacturer's instructions. The sequences of siRNAs for target as follows: FADD: 5′-CACAGAGAAGGAGAACGCA-3′; ATG5-#1: 5′-GC AACUCUGGAUGGGAUUGTT-3′; ATG5-#2: 5′-GACG TTGGTAACTGACAAATT-3′.

### Western blotting

Cells were lysed in ice-cold lysis buffer (20 mMTris-HCl (pH 7.5), 150 mM NaCl, 1%Triton-X 100, 1 mM EDTA and a protein inhibitor cocktail) for 30 min. The supernatant was boiled with Laemmli sample buffer for SDS-PAGE. Antibodies as follows: anti-Rheb and anti-FADD from Abcam, anti-LC3B, anti-SQSTM1/p62, anti-p70s6k, anti-phospho-p70s6k (Thr389) and anti-ATG5 from Cell Signaling Tech, and other antibodies: anti-α-Tubulin (Epitomics, 2871-1), anti-Flag (Sigma-Aldrich, F7425), anti-GAPDH (Santa Cruz Biotechnology, L-3113). Band intensity was quantified by ChemiAnalysi software (Bioshine, China).

### Quantitative real-time PCR

Total RNA was extracted with TRIzol reagent (Invitrogen, USA) following the manufacturer's instructions. Quantitative real-time PCR was performed using reverse transcription kit (Takara, Japan) and SYBR Green PCR Master Mix (Roche, Germany). The primers as follows: Rheb, 5′-GCCGCCGATCACAGCAGCAGGAG-3′ and 5′-CCCACAGACCGGTAGCCCAGGAT-3′; FADD, 5′-GC CGCGCCTGGGGAAGAAGAC-3′ and 5′-GCAAAGCA GCGGCCCATCAGGA-3′; β-actin, 5′- CATCGAGCAC GGCATCGTCA-3′ and 5′-TAGCACAGCCTGGATAGC AAC-3′.

### Luciferase reporter assay

MCF-7 cells were transfected with siRNA/NC firstly and then cotransfected with Rheb-promoter luciferase and control pRL reporter for 24 h. Luciferase activities were measured consecutively by using Dual-Luciferase assays (Promega, USA). All measurements were normalized for Renilla luciferase activity to correct the variations in transfection efficiencies.

### LC-MS/MS analysis and bioinformatics analysis

The LC-MS/MS analysis was performed as previously described [37]. Protein samples were carried out using the bicinchoninic acid (BCA) method. Equal amount of protein (200 μg) was used for iTRAQ labeling according to the manufacturer's instructions. Raw MS/MS data were analyzed by the Agilent G2721AA Spectrum Mill MS Proteomics Workbench (Rev A.03.03.078) in the UniProtKB/SWISS Prot database for protein identification. The network building tool MetaCoreTM version 5.4 (GeneGo) was used to establish potential signaling network.

### Tissue microarray analysis

Tissue microarray (TMA) of breast cancer was purchased from Shanghai Outdo Biotech Co. Ltd. Specimens included stage II or III invasive ductal cancer (n=30) and adjacent normal tissue (n=30). TMA immunohistochemical analysis was performed as previously described [38]. The quantitative analysis of FADD and Rheb staining was applied with Image-Pro Plus software.

### Transmission electron microscopy assay

MCF-7 cells were cultured in 100 mm dishes and co-transfected with FADD siRNA or NC siRNA and Rheb expression vector. After 48 h, cells were harvested and washed with cold PBS once in a 1.5 ml microcentrifuge tube. Cells were fixed with 0.25% glutaraldehyde at 4°C overnight. Then samples were observed under transmission electron microscopy (Hitachi, Japan).

### Real-time cell analysis (RTCA) of cell proliferation

The procedure was described previously [39]. Briefly, cells were digested and counted with Automated Cell Counter (Invitrogen, USA). 5,000 cells of each group were seeded to modified 16-well plates (E-plate, Roche, Germany) and monitored using the xCELLigence RTCA DP instrument (Roche, Germany). Data collecting and analysis was in accordance with the manufacturer's guidelines.

### Statistical analysis

Data were presented as means–SD. Comparisons within groups were done with a t-test with repeated measures; p-values indicated in figures are <0.05 (*), <0.01(**), and <0.001 (***).

## SUPPLEMENTARY FIGURES AND TABLES



## Abbreviations

FADD: Fas-associated death domain-containing protein
Rheb: Ras homolog enriched in brain
LC-MS/MS: liquid chromatography linked to tandem mass spectrometry
mTORC1/2: mechanistic target of rapamycin complex 1/2
p70s6k: ribosomal S6 protein kinase, 70kD
PCD: programmed cell death
ATG: autophagy-related protein
GAPDH: glyceraldehyde-3-phosphate dehydrogenase
LC3: microtubule-associated protein 1 light chain 3
Act D: actinomycin D
CQ: chloroquine
NC: negative control
qRT-PCR: quantitative real-time PCR
TMA: tissue microarray
RCTA: real-time cell analysis
TEM: transmission electron microscopy
BCA: bicinchoninic acid.
