# Supplementary material for: Fas-associated protein with death domain (FADD) regulates autophagy through promoting the expression of Ras homolog enriched in brain (Rheb) in human breast adenocarcinoma cells
Source: Oncotarget. 2016 Mar 22;7(17):24572–84. doi: 10.18632/oncotarget.8249 (PMC5029724; doi:10.18632/oncotarget.8249)
Supplement: Supplementary file 1 [file oncotarget-07-24572-s001.pdf]

SUPPLEMENTARY FIGURES AND TABLE

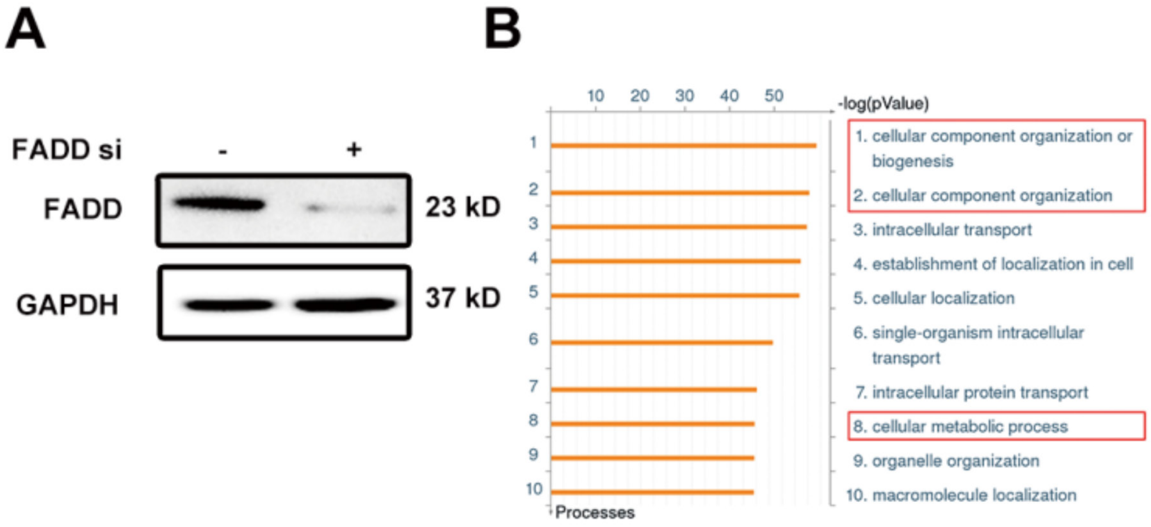

Supplementary Figure S1: Enrichment analysis of differentially expressed proteins in control and FADD-knockdown MCF-7 cells by GeneGo MetaCore. A. Validation of FADD expression. MCF-7 cells were transfected with 80 µM FADD siRNA/NC and then subjected to western blotting. **B.** Top ten most significant pathways predicted through GeneGo process analysis. The results were ordered by -log10 of the *p* value of the hypergeometric distribution.

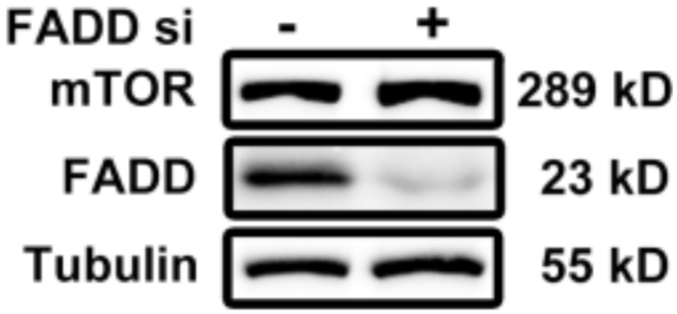

Supplementary Figure S2: FADD interference had no effect on mTOR expression. MCF-7 cells were transfected with 80 µM FADD siRNA or NC siRNA for 48 h. Cell lysates were detected with indicated antibodies by western blotting. Representative images were shown from 3 independent experiments.

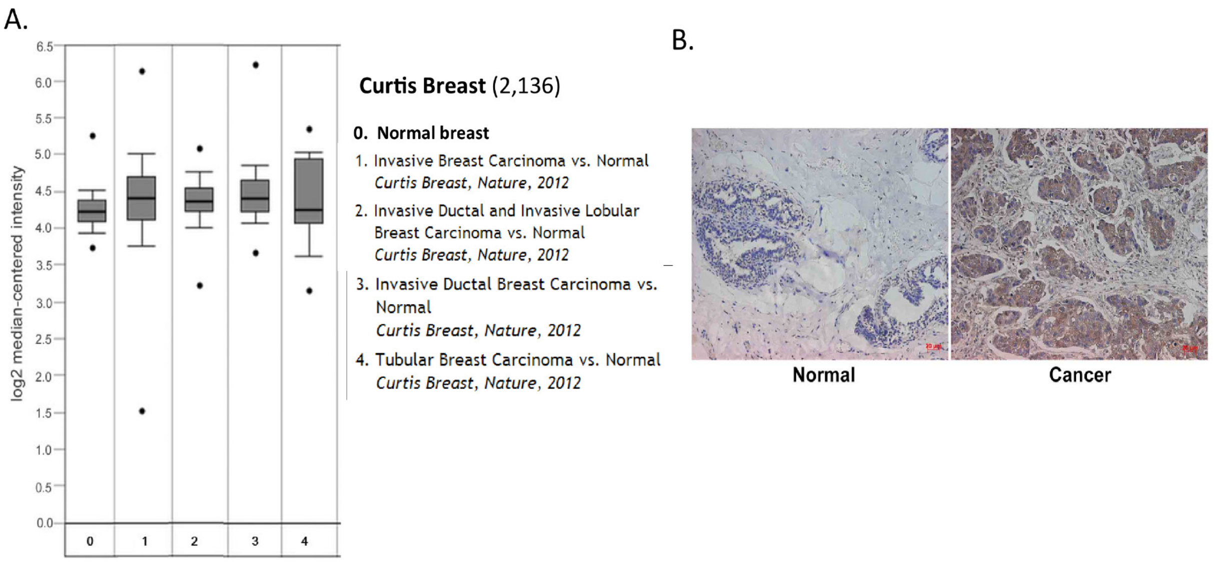

Supplementary Figure S3: Rheb overexpression is associated with human breast cancer progression. A. Increased Rheb mRNA expression was analyzed in Curtis Breast dataset with 2,136 samples. **B.** Tissue microarray (TMA) analysis for relative Rheb expression in human breast tumor tissue and adjacent histologically normal tissue. Representative images were shown.

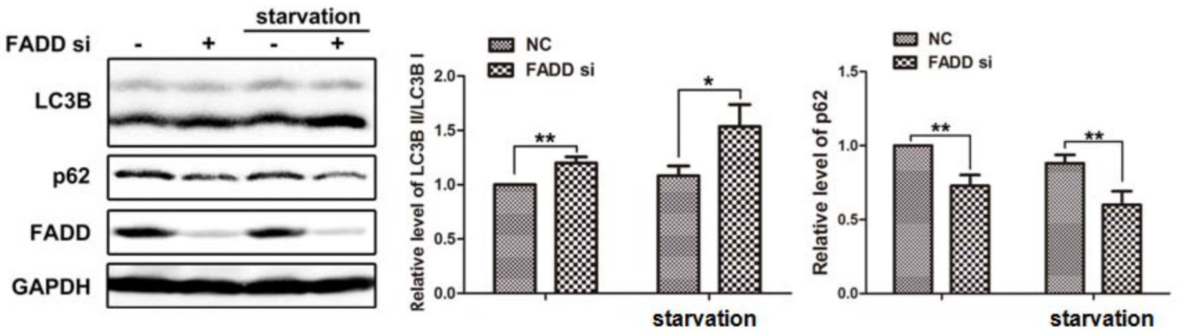

Supplementary Figure S4: FADD interference induced autophagy. MCF-7 cells were transfected with FADD siRNA/NC for 24 h and then starved with medium without serum for another 24 h. Proteins were detected with indicated antibodies by western blotting. Band intensity was quantified by chemiAnalysi software. Each bar is the mean of 3 independent experiments. Data are represented as mean ± S.D. $*p < 0.05$; $**p < 0.01$.

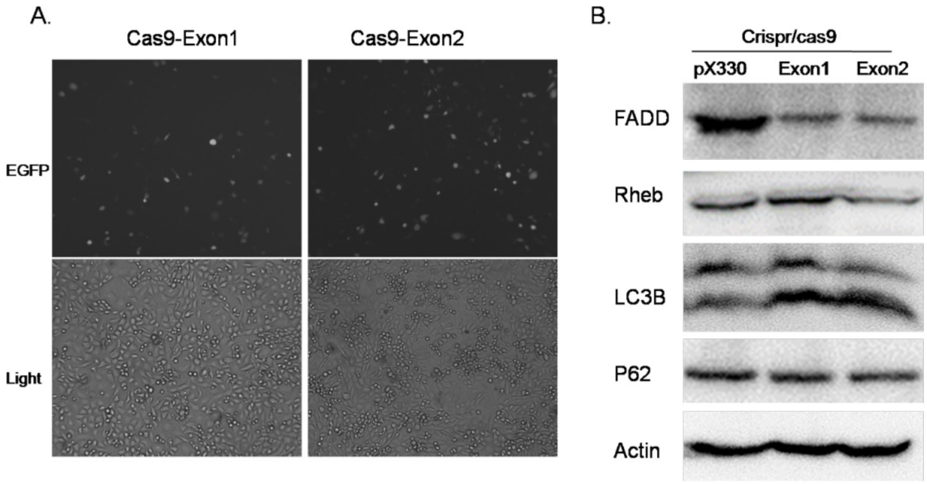

Supplementary Figure S5: FADD interference using Crispr/Cas9 system downregulated Rheb expression and induced autophagy. A. MCF-7 cells was transfected with Cas9-FADD construct targeting exon1 or exon2 respectively. EGFP indicated transfection efficiency. **B.** Cells harvested from (A) were lysed for western blot with indicated antibodies.

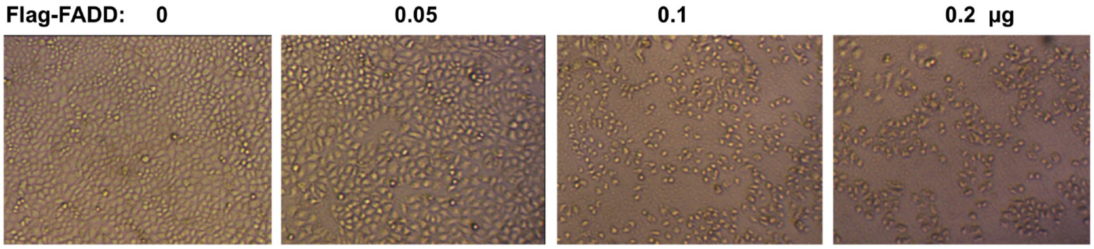

Supplementary Figure S6: FADD overexpression resulted in apoptosis. MCF-7 cells were transfected with Flag-FADD expression vector for 24h and the cell morphology was taken picture.

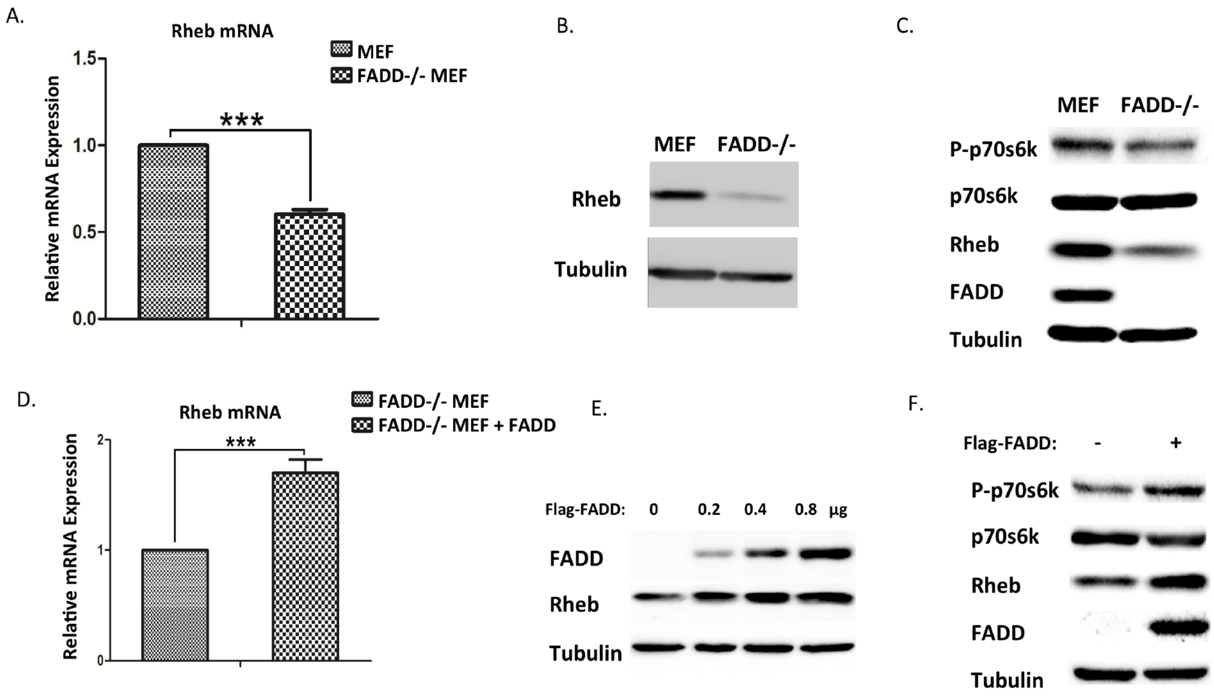

Supplementary Figure S7: FADD regulated Rheb expression. **A.** qPCR for Rheb mRNA in FADD-knockout MEF cells. **B.** Western blot analysis for Rheb protein level in FADD-knockout MEF cells. **C.** MEFs was lysated for detection as indicated by western blot. **D.** qPCR for Rheb mRNA in FADD-knockout MEFs with recovering FADD expression. **E.** FADD-/-MEFs were transfected with an increasing Flag-FADD expression vector for 24h and lysed for western blot analysis. **F.** FADD expression upregulated mTOR activity.

Supplementary Table S1: Deregulated proteins involved in Rheb-mTORC1 pathway

Protein Name	Network Objects	Accession	Fold Change
mechanistic target of rapamycin (serine/threonine kinase)	mTOR	P42345	-1.64437
Ras homolog enriched in brain	RHEB2	Q15382	-2.02643
